# Development and temporal validation of preoperative and combined preoperative–pathological models for lymph node metastasis in early gastric cancer

**DOI:** 10.3389/fonc.2026.1871868

**Published:** 2026-09-11

**Authors:** Jin Zhang, Lanyu Cai, Yunfei Kong, Zhongda Chen

**Affiliations:** 1 Department of General Surgery, Affiliated Hospital of Jiangsu University, Zhenjiang, Jiangsu, China; 2 Emergency Department, Xuancheng People’s Hospital, Xuancheng, Anhui, China

**Keywords:** early gastric cancer, endoscopic submucosal dissection, lymph node metastasis, nomogram, pathological factors, preoperative model, temporal validation

## Abstract

**Aim:**

To develop and temporally validate preoperative and combined preoperative–pathological models for lymph node metastasis (LNM) in early gastric cancer (EGC).

**Methods:**

We retrospectively analyzed 501 patients with EGC. Patients treated from January 2014 to December 2021 formed the training cohort (n = 347; 39 LNM events), and those treated from January 2022 to June 2026 formed the temporal validation cohort (n = 154; 21 LNM events). All preprocessing, exploratory LCR cut-off derivation, variable selection, coefficient estimation, and model choice were performed in the training cohort. The locked models were then evaluated without refitting in the temporal validation cohort. Performance was assessed using area under the receiver operating characteristic curve (AUC), Brier score, calibration intercept and slope, decision curve analysis, continuous net reclassification improvement (NRI), and integrated discrimination improvement (IDI). Conditional internal validation used 1,000 bootstrap samples with the final predictor sets held fixed.

**Results:**

The final preoperative model (PSL) included granulocyte count, albumin, LCR, tumor location, endoscopic ulceration, and long diameter. The combined model (Post-LR) included LCR, neurovascular invasion, long diameter, pathological ulceration, and differentiation. In temporal validation, the AUC was 0.730 (95% CI, 0.627–0.834) for PSL and 0.827 (95% CI, 0.723–0.932) for Post-LR. The corresponding Brier scores were 0.119 and 0.081. Compared with the pathology-only model, Post-LR had no statistically significant incremental benefit in temporal validation. An exploratory training-derived LCR cut-off of 2.20 separated risk in the training cohort but not in the temporal validation cohort.

**Conclusion:**

The preoperative and combined models provide complementary, hypothesis-generating risk estimates at different clinical stages. They should be used only as adjuncts to established ESD criteria and multidisciplinary judgment. External validation in prospective, real-world ESD cohorts is required before clinical implementation.

## 1 Introduction

Cardiovascular diseases and cancer are the leading causes of premature mortality worldwide. As living environments and dietary habits evolve, cancer has gradually surpassed cardiovascular disease as a principal driver of death (1). Among all malignancies, gastric cancer ranks as the fifth most common tumor globally and represents the fourth leading cause of cancer-related death in men and the fifth in women (2).

Early gastric cancer (EGC) is defined as carcinoma confined to the mucosal or submucosal layer, irrespective of lymph node metastasis (LNM) status (3, 4). Treatment selection depends strongly on the estimated risk of nodal disease. The Japanese Gastric Cancer Association guideline recommends endoscopic resection only when the likelihood of LNM is negligible; otherwise, gastrectomy with appropriate lymphadenectomy is considered (5). Endoscopic submucosal dissection (ESD) can achieve en bloc resection with less invasiveness than surgery in appropriately selected patients, and it generally provides better en bloc and complete resection rates than endoscopic mucosal resection (6–9). Nevertheless, ESD does not remove regional lymph nodes, so an underestimated LNM risk may lead to insufficient treatment.

Lymph nodes serve as critical hubs for metastatic cell filtration, immune regulation, and secondary distant dissemination, rendering LNM an important prognostic marker across numerous malignancies. Wang et al. (10) employed deep learning techniques to analyze histopathological images of resected lymph nodes, underscoring the pivotal role of lymph node status in gastric cancer prognosis. Seto et al. (11). directly addressed the impact of lymph node spread on survival in EGC, emphasizing the prognostic significance of LNM. Ji et al. (12) comprehensively reviewed the molecular mechanisms, clinical implications, and therapeutic strategies of LNM across various anatomical sites, proposing that LNM serves not only as a significant indicator of gastric cancer progression but also as a pivotal determinant of treatment strategy and prognostic assessment. Liu et al. (13) further confirmed the substantial impact of LNM on survival outcomes in EGC through a retrospective cohort study. Lymphatic metastasis represents a key route of gastric cancer dissemination, and the presence or absence of nodal involvement has become a critical indicator for assessing patient survival and prognostic outcomes.

LNM prediction models have been widely applied in clinical practice, with numerous studies developing risk stratification tools based on EGC characteristics (14–16). Chen et al. (17) demonstrated that LNM in EGC is a key factor in evaluating prognosis and determining treatment strategies, suggesting that ESD or EMR should be considered when patients exhibit a low risk of LNM.

Accordingly, this study developed two complementary LNM prediction tools. The preoperative model uses information available before definitive treatment and is intended only to supplement—not replace—standard ESD eligibility criteria. The combined preoperative–pathological model is intended for risk re-evaluation after endoscopic resection, when pathological information becomes available and the need for additional gastrectomy is being considered. Because both models were derived from patients who underwent radical gastrectomy, their application to an ESD population remains provisional.

## 2 Methods

### 2.1 Participants

We retrospectively collected data from patients who underwent radical gastrectomy with lymphadenectomy at the Affiliated Hospital of Jiangsu University between January 2014 and June 2026. After applying the predefined criteria, 501 patients were included. Although the combined model is intended to inform post-ESD risk re-evaluation, it was derived from a radical-gastrectomy cohort in which nodal status was established by lymphadenectomy.

### 2.2 Inclusion criteria

(1) Postoperative pathological diagnosis of EGC according to World Health Organization histological criteria (2); Underwent radical gastrectomy with lymph node dissection (3); Complete blood test results available within one week before surgery (4); Underwent gastroscopy during hospitalization with clear and complete endoscopic images; and (5) Complete postoperative pathological dataset.

### 2.3 Exclusion criteria

**Figure 1:**
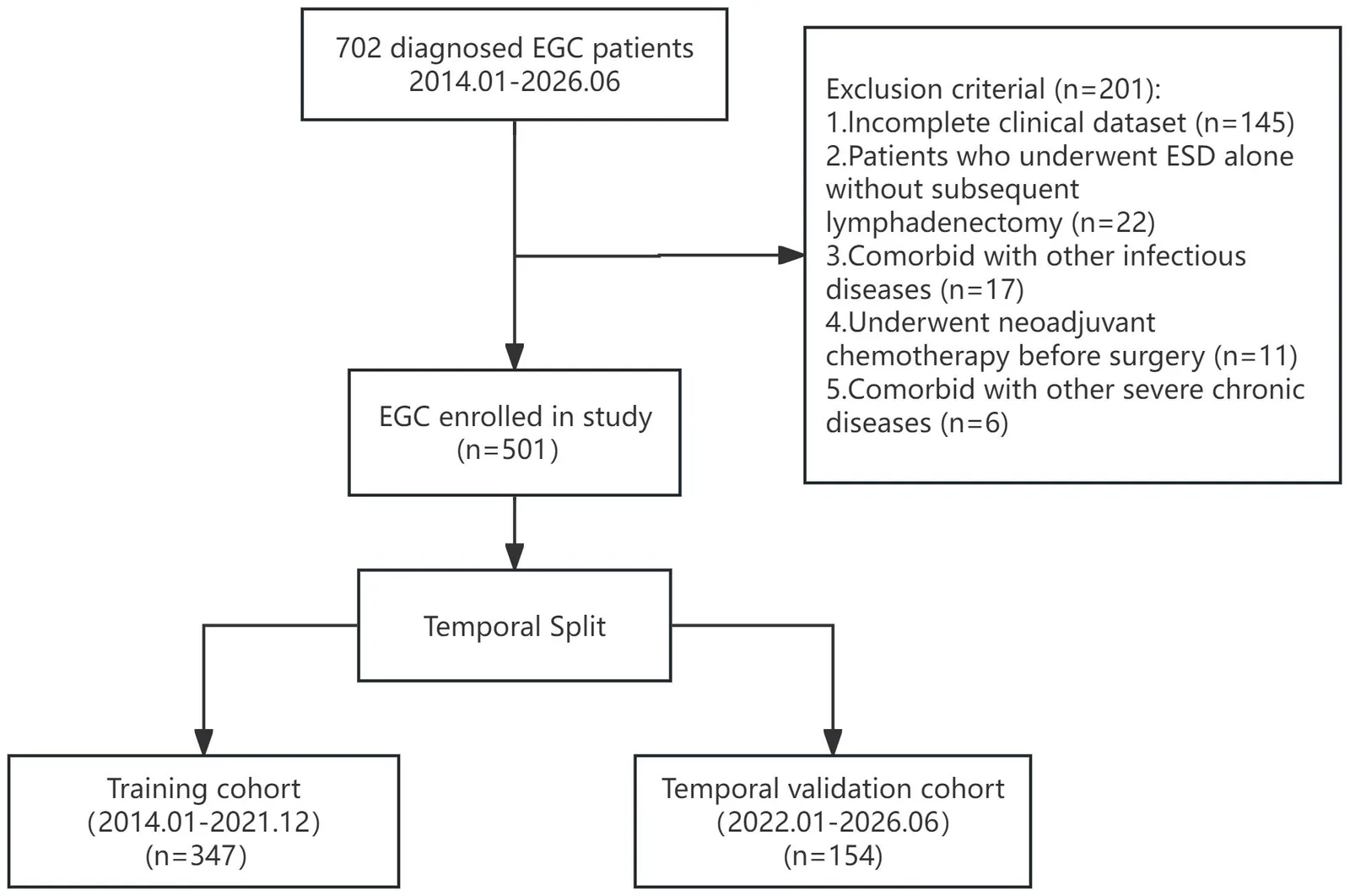
Flowchart of patient selection and temporal dataset split. Patients treated from January 2014 through December 2021 formed the training cohort (group = 1; n = 347), and those treated from January 2022 through June 2026 formed the temporal validation cohort (group = 2; n = 154).

(1) Incomplete clinical data (2); ESD alone without subsequent lymphadenectomy (3); active infectious disease (4); neoadjuvant chemotherapy before surgery; or (5) severe chronic comorbidity that could materially affect the laboratory indicators (**Figure 1**).

## 3 Ethics approval

This study was approved by the Ethics Review Board of the Affiliated Hospital of Jiangsu University (Approval number: KY2022K0910) and adhered to the principles of the Declaration of Helsinki.

## 4 Procedures

Clinical data were retrospectively extracted from the electronic inpatient record system. Preoperative variables comprised age, sex, lymphocyte count (LYC), granulocyte count (GR), platelet count (PLT), C-reactive protein (CRP), neutrophil-to-lymphocyte ratio (NLR), platelet-to-lymphocyte ratio (PLR), albumin (ALB), carcinoembryonic antigen (CEA), carbohydrate antigens 19-9, 125, and 72-4, ferritin (FER), fibrinogen (FIB), endoscopic ulceration, tumor location, and long and short tumor diameters. Blood measurements were obtained within one week before surgery. Pathological variables comprised neurovascular invasion, pathological depth (T1a/T1b), differentiation, pathological ulceration, and LNM status. Pathological depth and differentiation from the resection specimen were not eligible predictors for the preoperative model.

Patients treated from January 2014 to December 2021 were assigned to the training cohort (group = 1; n = 347), and patients treated from January 2022 to June 2026 were assigned to the temporal validation cohort (group = 2; n = 154). The temporal validation cohort remained locked during preprocessing, cut-off derivation, variable selection, coefficient estimation, and final model choice. After the final predictor sets and coefficients were fixed, the models were applied to group 2 without refitting or recalibration.

## 5 Definition and grouping of LCR

LCR was defined as LYC divided by CRP (18) and was recalculated from these two source variables for every patient before analysis. The derived LCR field in the supplied dataset was not used when it differed from the source-variable calculation. As an exploratory secondary analysis, candidate cut-offs from the 10th through the 90th percentiles of the training-cohort LCR distribution were scanned. For each candidate, a chi-square test compared LNM status between the low- and high-LCR groups; the cut-off with the smallest training-cohort P value was retained.

The training-derived cut-off was then applied unchanged to the temporal validation cohort. Because the scan involved multiple candidate thresholds and no multiplicity adjustment, the categorized LCR analysis was considered exploratory. Continuous LCR remained the primary representation in model development.

## 6 Statistical analysis

Continuous variables were summarized as mean (standard deviation) or median (interquartile range), as appropriate. Cohorts were compared using Student’s t test or the Mann–Whitney U test for continuous variables and the chi-square or Fisher exact test for categorical variables. All tests were two-sided, and P < 0.05 was considered statistically significant. Statistical analyses were performed using R version 4.3.3 (R Foundation for Statistical Computing, Vienna, Austria). The glmnet, pROC, rms, rmda, ResourceSelection, and boot packages were used for penalized regression, ROC and DeLong analyses, logistic modeling, calibration and nomograms, decision curve analysis, Hosmer–Lemeshow testing, and bootstrap resampling, as applicable. A fixed random seed (20260806) was used for resampling-based analyses.

All model development was confined to the training cohort. Two training-only selection strategies were examined in parallel: 10-fold cross-validated Lasso regression and stepwise logistic regression minimizing Akaike’s information criterion (AIC). Variables associated with LNM at P < 0.05 in training-cohort univariable analysis were eligible for the stepwise candidate models. Both the minimum-error and one-standard-error Lasso solutions were inspected. The simplified preoperative Lasso candidate (PSL) was defined by removing penalized coefficients with an absolute value below 0.05, followed by an unpenalized logistic refit of the retained predictor set. Categorical predictors were represented by indicator variables, with the upper third as the reference for tumor location and poor differentiation as the reference for differentiation. Final model choice was made using training-cohort fit, discrimination, reclassification, decision curves, parsimony, and clinical interpretability before the temporal validation results were examined.

Locked-model discrimination was summarized by AUC with 95% confidence intervals; pairwise AUC comparisons used DeLong’s test. Continuous NRI and IDI with 95% confidence intervals were estimated using 4,000 outcome-stratified bootstrap samples. Calibration was described using the Brier score, calibration intercept, calibration slope, grouped calibration plots, and the Hosmer–Lemeshow statistic. Decision curve analysis was evaluated over threshold probabilities from 0.01 to 0.40. Published predictor sets were re-estimated in the present training cohort and therefore served as indirect predictor-set benchmarks rather than validations of the original published equations.

Conditional internal validation of PSL and Post-LR used 1,000 bootstrap samples. In each bootstrap sample, the prespecified final predictor set was refitted, and optimism was estimated by comparing performance in the bootstrap sample with performance of that bootstrap-fitted model in the original training data. The variable-selection process itself was not repeated. The resulting optimism-corrected estimates therefore assess the fixed final model specifications but do not capture uncertainty introduced by data-driven variable selection.

## 7 Results

### 7.1 Study population and baseline characteristics

The training cohort contained 39 LNM-positive and 308 LNM-negative patients; the temporal validation cohort contained 21 LNM-positive and 133 LNM-negative patients. Baseline characteristics are summarized in **Table 1**. Stratified LNM incidence for key clinical and pathological factors is provided in **Supplementary Table S1**.

**Table 1:** Clinicopathological characteristics of the training and temporal validation cohorts.

Clinical factors	Training cohort (N = 347)		Temporal validation cohort (N = 154)		P
	LNM+(N = 39)	LNM-(N = 308)	LNM+(N = 21)	LNM-(N = 133)	0.540
Sex					0.158
Female	17(43.6%)	77(25.0%)	7(33.3%)	45(33.8%)	
Male	22(56.4%)	231(75.0%)	14(66.7%)	88(66.2%)	
Age	62(14.5)	64 (9)	65 (5)	64(8)	0.951
LYC	1.6(0.85)	1.7(0.7)	1.4(0.7)	1.7(0.7)	0.882
GR	3.1(1.55)	3.25(1.62)	3.1(1.5)	3.1(1.5)	0.153
PLT	199(58)	187.5(71)	189(73)	185(69)	0.804
CRP	1(0.9)	0.5(0.8)	0.5(0.8)	0.6(0.8)	0.875
NLR	1.77(1.14)	2(1.26)	1.81(1.35)	1.78(1.12)	0.360
PLR	112.14(74.39)	108.53(56.8)	113.33(113.25)	111.2(60.65)	0.975
LCR	1.7(1.41)	3.15(6.6)	2.4(2)	2.4(7.2)	0.797
ALB	39.9(3.6)	40.35(4.85)	38.3(5.4)	39.5(4.9)	0.428
CEA	2.4(2.1)	2.4(1.6)	2.97(2.4)	2.32(1.81)	0.116
CA19-9	6.1(10.45)	5.25(6.9)	4.5(6.2)	5.25(9.5)	0.468
CA125	10.1(4.25)	10.1(5.52)	10.1(4.2)	9.7(4.8)	0.266
CA72-4	1.82(0.99)	1.68(1.29)	1.97(2.34)	2.2(2.33)	0.523
FER	127.4(108.75)	127.4(105.1)	149.29(3.8)	149.29(74.84)	0.809
FIB	2.56(0.65)	2.54(0.8)	2.48(0.5)	2.52(0.73)	0.376
Tumor location					0.176
Upper third	6(15.4%)	117(38.0%)	3(14.3%)	40(30.08%)	
Middle third	7(17.9%)	72(23.4%)	2(9.5%)	32(24.06%)	
Lower third	26(66.7%)	119(38.6%)	16(76.2%)	61(45.86%)	
Long diameter	2.5(1.1)	1.8(1.1)	2(1.1)	1.8(1.3)	0.935
Short diameter	1.7(1)	1.15(0.7)	1(1.2)	1.2(0.7)	0.830
Endoscopic ulceration					0.597
No	12(30.8%)	194(63.0%)	10(47.6%)	86(64.7%)	
Yes	27(69.2%)	114(37.0%)	11(52.4%)	47(35.3%)	
Neurovascular invasion					0.306
No	12(30.8%)	288(93.5%)	8(38.1%)	119(89.5%)	
Yes	27(69.2%)	20(6.5%)	13(61.9%)	14(10.5%)	
Tumor depth					0.996
T1a	8(20.5%)	139(45.1%)	2(9.5%)	64(48.1%)	
T1b	31(79.5%)	169(54.9%)	19(90.5%)	69(51.9%)	
Pathological ulceration					0.565
No	10(25.6%)	186(60.4%)	9(42.9%)	73(54.9%)	
Yes	29(74.4%)	122(39.6%)	12(57.1%)	60(45.1%)	
Differentiation					0.810
Poor	26(66.7%)	97(31.5%)	16(76.2%)	34(25.6%)	
Moderate	10(25.6%)	107(34.7%)	5(23.8%)	49(36.8%)	
Well	3(7.7%)	104(33.8%)	0(0.0%)	50(37.6%)	

Values are n (%) or median (interquartile range). P values compare the overall training and temporal validation cohorts. LNM, lymph node metastasis; LYC, lymphocyte count; GR, granulocyte count; PLT, platelet count; CRP, C-reactive protein; NLR, neutrophil-to-lymphocyte ratio; PLR, platelet-to-lymphocyte ratio; LCR, lymphocyte-to-C-reactive protein ratio; ALB, albumin; FIB, fibrinogen; FER, ferritin.

### 7.2 Training-only development of preoperative and combined candidate models

**Figure 2:**
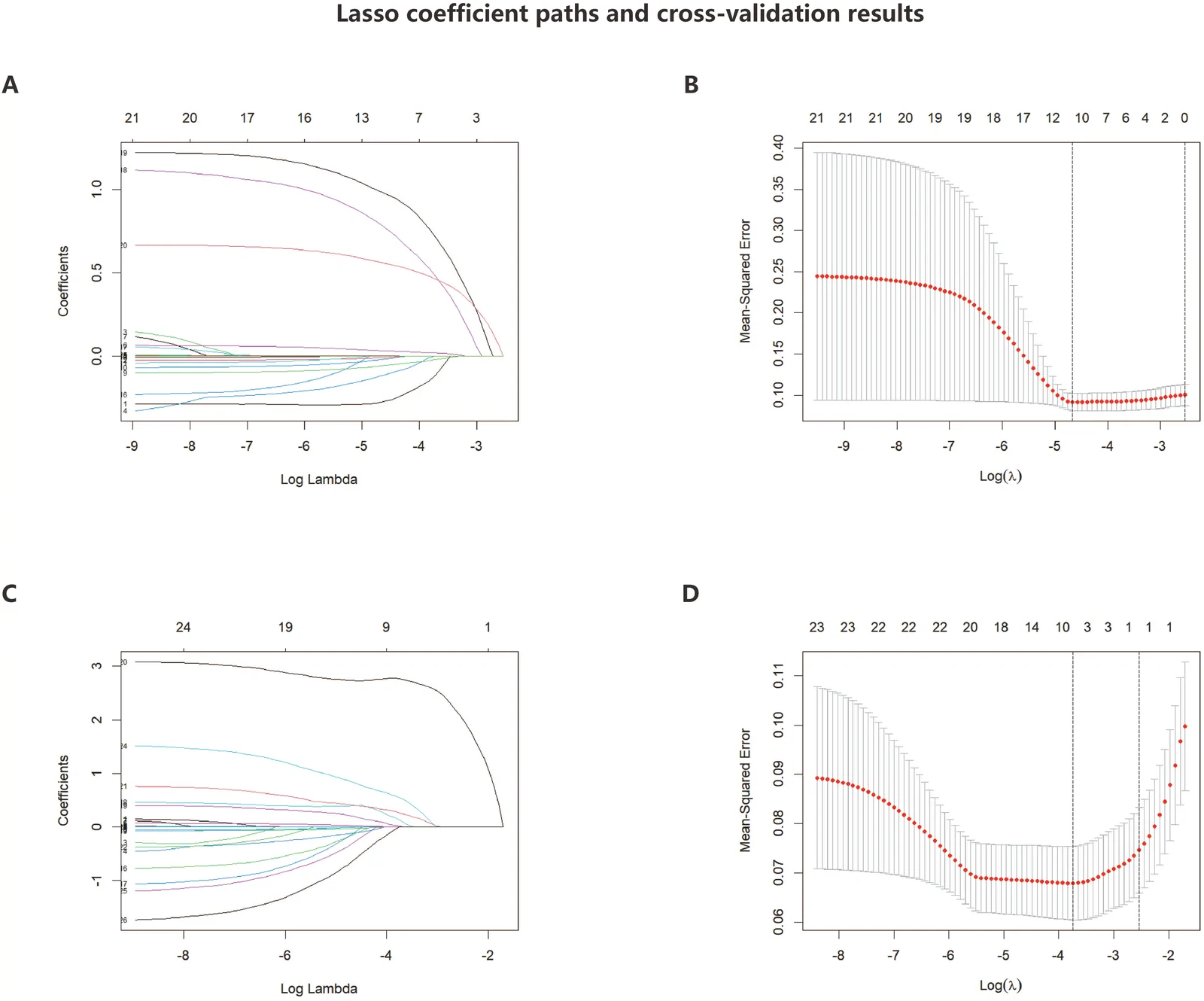
Lasso coefficient paths and cross-validation results. Training-cohort Lasso analyses for preoperative candidates **(A, B)** and combined preoperative–pathological candidates **(C, D)**. Coefficient paths are shown in **(A, C)**; 10-fold cross-validation curves are shown in **(B, D)**. The minimum-error solution was used when the one-standard-error solution retained only one or no predictor.

For preoperative prediction, the minimum-AIC logistic model (PLR; AIC = 208.94) retained continuous LCR, tumor location, endoscopic ulceration, and long diameter (**Table 2**). In parallel, 10-fold cross-validated Lasso regression was performed in the training cohort (**Figures 2A, B**). The one-standard-error solution retained only one variable; therefore, the minimum-error solution was used as the fuller Lasso candidate (PL). That solution retained sex, age, GR, PLT, CRP, PLR, ALB, LCR, tumor location, endoscopic ulceration, and long diameter.

**Table 2:** Univariable and minimum-AIC multivariable logistic regression for preoperative predictors in the training cohort.

Variable	Univariable OR	95% CI	P	Multivariable OR	95% CI	P
Sex						
Female	1.00	Reference				
Male	0.43	0.22–0.85	0.016			
Age	0.98	0.95–1.02	0.338			
LYC	0.97	0.53–1.77	0.918			
GR	0.80	0.59–1.07	0.133			
PLT	1.00	1.00–1.01	0.487			
CRP	1.10	1.00–1.21	0.060			
NLR	0.84	0.60–1.18	0.316			
PLR	1.00	1.00–1.01	0.411			
LCR	0.91	0.84–0.99	0.023	0.91	0.83–0.99	0.026
ALB	0.94	0.86–1.03	0.196			
CEA	1.00	0.86–1.16	0.983			
CA19-9	1.02	0.99–1.06	0.230			
CA125	1.00	0.96–1.05	0.939			
CA72-4	0.99	0.91–1.06	0.702			
FER	1.00	0.99–1.00	0.329			
FIB	1.18	0.68–2.05	0.548			
Tumor location						
Upper third	1.00	Reference		1.00	Reference	
Middle third	1.90	0.61–5.86	0.267	1.29	0.39–4.28	0.677
Lower third	4.26	1.69–10.72	0.002	3.04	1.14–8.08	0.026
Short diameter	2.03	1.36–3.04	0.001			
Long diameter	2.08	1.50–2.88	<0.001	1.98	1.39–2.82	<0.001
Endoscopic ulceration						
No	1.00	Reference		1.00	Reference	
Yes	3.83	1.87–7.85	<0.001	3.58	1.64–7.82	0.001

The multivariable columns report the minimum-AIC preoperative logistic model (PLR). All candidate predictors were available before treatment; pathological depth and differentiation were excluded. OR, odds ratio; CI, confidence interval.

For clinical parsimony, penalized coefficients with an absolute value below 0.05 were removed from the fuller Lasso solution. The resulting six-predictor set—GR, ALB, continuous LCR, tumor location, endoscopic ulceration, and long diameter—was refitted by logistic regression and designated PSL. This coefficient threshold was an exploratory simplification rule rather than a significance test.

Thus, three preoperative candidates were specified in the training cohort: PLR, the minimum-error Lasso predictor set refitted by logistic regression (PL), and the simplified predictor set refitted by logistic regression (PSL). Their specifications were fixed before temporal validation.

For the combined preoperative–pathological setting, the minimum-AIC logistic model (Post-LR; AIC = 154.85) retained continuous LCR, neurovascular invasion, long diameter, pathological ulceration, and differentiation (**Table 3**). The minimum-error Lasso predictor set, refitted by logistic regression as Post-Lasso (AIC = 159.64), retained neurovascular invasion, long diameter, pathological ulceration, tumor location, and endoscopic ulceration (**Figures 2C, D**). Both candidate models therefore included variables available before treatment and variables obtained from the resection specimen; neither was a pathology-only model.

**Table 3:** Univariable and minimum-AIC multivariable logistic regression for the combined preoperative–pathological model in the training cohort.

Variable	Univariable OR	95% CI	P	Multivariable OR	95% CI	P
Sex						
Female	1.00	Reference				
Male	0.43	0.22–0.85	0.016			
Age	0.98	0.95–1.02	0.338			
LYC	0.97	0.53–1.77	0.918			
GR	0.80	0.59–1.07	0.133			
PLT	1.00	1.00–1.01	0.487			
CRP	1.10	1.00–1.21	0.060			
NLR	0.84	0.60–1.18	0.316			
PLR	1.00	1.00–1.01	0.411			
LCR	0.91	0.84–0.99	0.023	0.94	0.86–1.03	0.157
ALB	0.94	0.86–1.03	0.196			
CEA	1.00	0.86–1.16	0.983			
CA19-9	1.02	0.99–1.06	0.230			
CA125	1.00	0.96–1.05	0.939			
CA72-4	0.99	0.91–1.06	0.702			
FER	1.00	0.99–1.00	0.329			
FIB	1.18	0.68–2.05	0.548			
Tumor location						
Upper third	1.00	Reference				
Middle third	1.90	0.61–5.86	0.267			
Lower third	4.26	1.69–10.72	0.002			
Endoscopic ulceration						
No	1.00	Reference				
Yes	3.83	1.87–7.85	<0.001			
Neurovascular invasion						
No	1.00	Reference		1.00	Reference	
Yes	32.40	14.31–73.37	<0.001	16.56	6.78–40.49	<0.001
Short diameter	2.03	1.36–3.04	0.001			
Long diameter	2.08	1.50–2.88	<0.001	1.76	1.15–2.71	0.009
Tumor depth						
T1a	1.00	Reference				
T1b	3.19	1.42–7.16	0.005			
Pathological ulceration						
No	1.00	Reference		1.00	Reference	
Yes	4.42	2.08–9.40	<0.001	3.99	1.54–10.29	0.004
Differentiation						
Poor	1.00	Reference		1.00	Reference	
Moderate	0.35	0.16–0.76	0.008	0.44	0.16–1.18	0.103
Well	0.11	0.03–0.37	<0.001	0.22	0.05–0.93	0.039

The multivariable columns report Post-LR, which was selected in the training cohort from both preoperative and pathological candidate variables. Poor differentiation is the reference category. OR, odds ratio; CI, confidence interval.

### 7.3 Development of a preoperative prediction model for LNM in EGC

**Figure 3:**
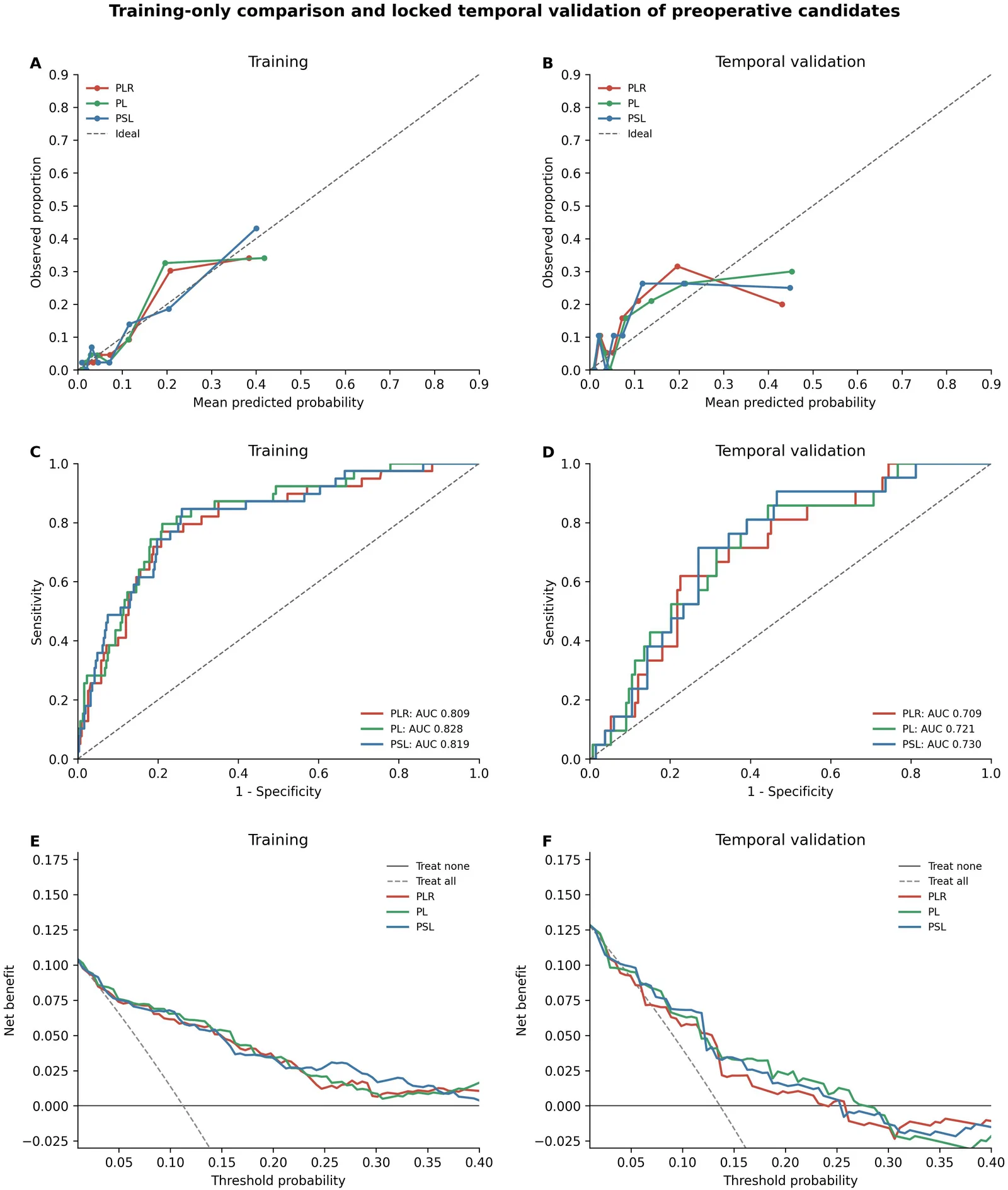
Training-only comparison and locked temporal validation of the preoperative candidate models. PL, PLR, and PSL were specified and compared in the training cohort before temporal validation. **(A, B)** show calibration, **(C, D)** show ROC curves, and **(E, F)** show decision curves in the training and temporal validation cohorts, respectively.

In the training cohort, Brier scores were 0.084 for PLR, 0.081 for PL, and 0.082 for PSL; the corresponding Hosmer–Lemeshow P values were 0.907, 0.545, and 0.217. When the locked models were applied to the temporal validation cohort, Brier scores were 0.120, 0.121, and 0.119. Calibration slopes were 0.518, 0.511, and 0.544, respectively, indicating overfitting and weaker calibration in the later cohort (**Table 4**; **Figures 3A, B**).

**Table 4:** Fit and calibration of candidate models and predictor-set benchmarks.

Model	Cohort	AIC	Brier	Calibration intercept	Calibration slope	HL P
Preoperative candidate models						
PLR	Training	208.94	0.084	-0.000	1.000	0.907
PLR	Temporal validation	—	0.120	-0.658	0.518	0.006
PL	Training	214.45	0.081	-0.000	1.000	0.545
PL	Temporal validation	—	0.121	-0.698	0.511	<0.001
PSL	Training	209.27	0.082	-0.000	1.000	0.217
PSL	Temporal validation	—	0.119	-0.625	0.544	0.011
Combined candidate models						
Post-LR	Training	154.85	0.055	-0.000	1.000	0.335
Post-LR	Temporal validation	—	0.081	-0.079	0.783	0.085
Post-Lasso	Training	159.64	0.056	0.000	1.000	0.124
Post-Lasso	Temporal validation	—	0.097	-0.401	0.655	0.006
Predictor-set benchmarks						
Post-LR	Training	154.85	0.055	-0.000	1.000	0.335
Post-LR	Temporal validation	—	0.081	-0.079	0.783	0.085
Li predictor-set benchmark	Training	172.36	0.064	0.000	1.000	0.798
Li predictor-set benchmark	Temporal validation	—	0.097	-0.517	0.677	0.039
Lin predictor-set benchmark	Training	210.62	0.083	-0.000	1.000	0.827
Lin predictor-set benchmark	Temporal validation	—	0.100	0.381	1.042	0.170

AIC is reported only for the training-cohort refit. Validation predictions were produced from locked training coefficients without refitting. Ideal calibration has intercept 0 and slope 1. HL, Hosmer–Lemeshow test; Brier, Brier score.

Training-cohort AUCs were 0.828 (95% CI, 0.761–0.894) for PL, 0.809 (95% CI, 0.736–0.882) for PLR, and 0.819 (95% CI, 0.748–0.890) for PSL. In the temporal validation cohort, the corresponding AUCs were 0.721 (95% CI, 0.612–0.829), 0.709 (95% CI, 0.601–0.817), and 0.730 (95% CI, 0.627–0.834). No pairwise AUC difference was statistically significant. In the training cohort, PSL improved continuous NRI versus PLR (0.405; 95% CI, 0.102–0.720; P = 0.009), but its IDI difference was not significant. Neither NRI nor IDI improvement was confirmed in the temporal validation cohort (**Table 5**; **Figures 3C, D**).

**Table 5:** Pairwise discrimination and reclassification comparisons.

Comparison	Cohort	AUC	AUC P	Continuous NRI (95% CI)	P	IDI (95% CI)	P
Preoperative candidates							
PL vs PLR	Training	0.828 vs 0.809	0.153	0.323 (-0.011 to 0.638)	0.055	0.035 (-0.002 to 0.074)	0.075
PSL vs PLR	Training	0.819 vs 0.809	0.363	0.405 (0.102 to 0.720)	0.009	0.015 (-0.009 to 0.038)	0.225
PSL vs PL	Training	0.819 vs 0.828	0.320	-0.298 (-0.631 to 0.042)	0.088	-0.020 (-0.047 to 0.005)	0.113
PL vs PLR	Temporal validation	0.721 vs 0.709	0.697	0.135 (-0.321 to 0.586)	0.579	0.010 (-0.032 to 0.050)	0.646
PSL vs PLR	Temporal validation	0.730 vs 0.709	0.294	-0.015 (-0.476 to 0.431)	0.962	0.011 (-0.009 to 0.030)	0.267
PSL vs PL	Temporal validation	0.730 vs 0.721	0.651	-0.160 (-0.612 to 0.311)	0.510	0.001 (-0.037 to 0.042)	0.996
Combined candidates							
Post-LR vs Post-Lasso	Training	0.897 vs 0.881	0.446	0.143 (-0.203 to 0.470)	0.424	0.014 (-0.025 to 0.050)	0.477
Post-LR vs Post-Lasso	Temporal validation	0.827 vs 0.798	0.315	0.627 (0.185 to 1.033)	0.008	0.045 (0.008 to 0.081)	0.015
Predictor-set benchmarks							
Post-LR vs Li benchmark	Training	0.897 vs 0.865	0.234	0.582 (0.250 to 0.903)	<0.001	0.074 (0.025 to 0.127)	0.006
Post-LR vs Lin benchmark	Training	0.897 vs 0.796	0.005	1.033 (0.730 to 1.309)	<0.001	0.275 (0.195 to 0.356)	<0.001
Post-LR vs Li benchmark	Temporal validation	0.827 vs 0.773	0.307	0.306 (-0.165 to 0.747)	0.204	0.019 (-0.042 to 0.081)	0.555
Post-LR vs Lin benchmark	Temporal validation	0.827 vs 0.814	0.785	0.712 (0.276 to 1.123)	0.003	0.187 (0.060 to 0.308)	0.002

AUC comparisons used DeLong’s test. Continuous NRI and IDI confidence intervals and P values were estimated using 4,000 outcome-stratified bootstrap samples. The temporal validation results were not used to select the final model. AUC, area under the curve; NRI, net reclassification improvement; IDI, integrated discrimination improvement; CI, confidence interval.

PSL was selected before temporal validation because it offered a compact six-variable specification, discrimination similar to the fuller candidates, a training-cohort NRI improvement over PLR, and favorable training-cohort decision curves. Temporal validation was then used only to estimate out-of-time performance. The lower calibration slope and significant Hosmer–Lemeshow result in group 2 show that PSL requires recalibration and external validation before clinical use (**Figures 3E, F**).

### 7.4 Development of the combined preoperative–pathological model

**Figure 4:**
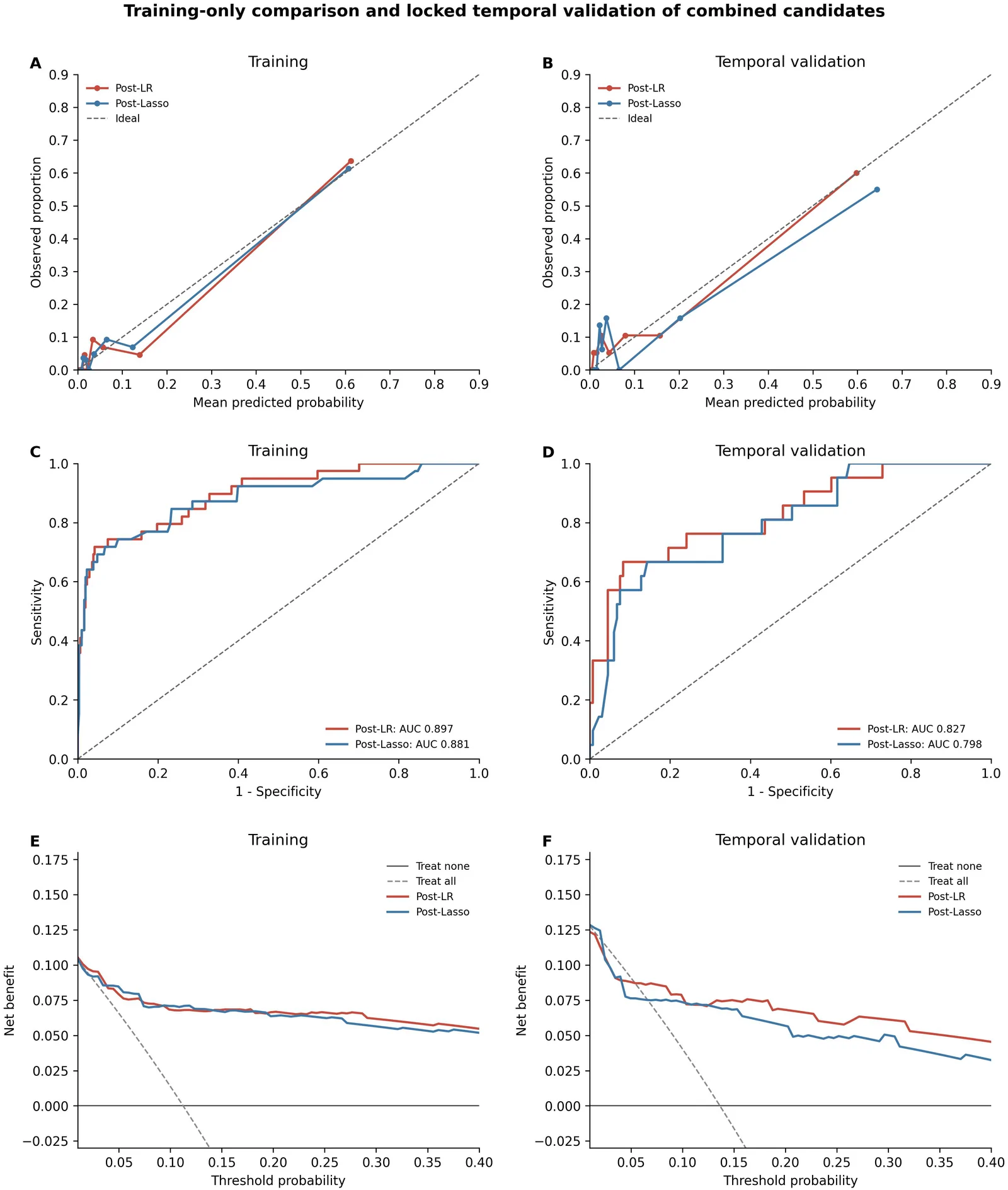
Training-only comparison and locked temporal validation of combined candidate models. Post-LR and Post-Lasso were specified and compared in the training cohort. **(A, B)** show calibration, **(C, D)** show ROC curves, and **(E, F)** show decision curves in the training and temporal validation cohorts, respectively.

Post-LR and Post-Lasso were compared in the training cohort before the temporal validation data were examined. Post-LR had a lower AIC (154.85 vs 159.64), a slightly lower Brier score (0.0546 vs 0.0560), and similar training-cohort AUC (0.897 vs 0.881; P = 0.446). The continuous NRI and IDI differences between the candidates were not statistically significant in the training cohort. These results, together with parsimony and clinical interpretability, supported selection of Post-LR as the final combined model (**Tables 4**, **5**; **Figure 4**).

After selection, the locked Post-LR and Post-Lasso models were applied to the temporal validation cohort. Post-LR had an AUC of 0.827 (95% CI, 0.723–0.932) compared with 0.798 (95% CI, 0.694–0.902) for Post-Lasso (P = 0.315). The Brier scores were 0.081 and 0.097, and calibration slopes were 0.783 and 0.655, respectively. Continuous NRI (0.627; 95% CI, 0.185–1.033; P = 0.008) and IDI (0.045; 95% CI, 0.008–0.081; P = 0.015) favored Post-LR, although the limited number of validation events warrants cautious interpretation.

Post-LR was therefore retained as the final combined preoperative–pathological model. This choice was made from training-cohort evidence; the temporal validation results are reported as an out-of-time evaluation and were not used to alter the specification.

### 7.5 Nomogram development and application

**Figure 5:**
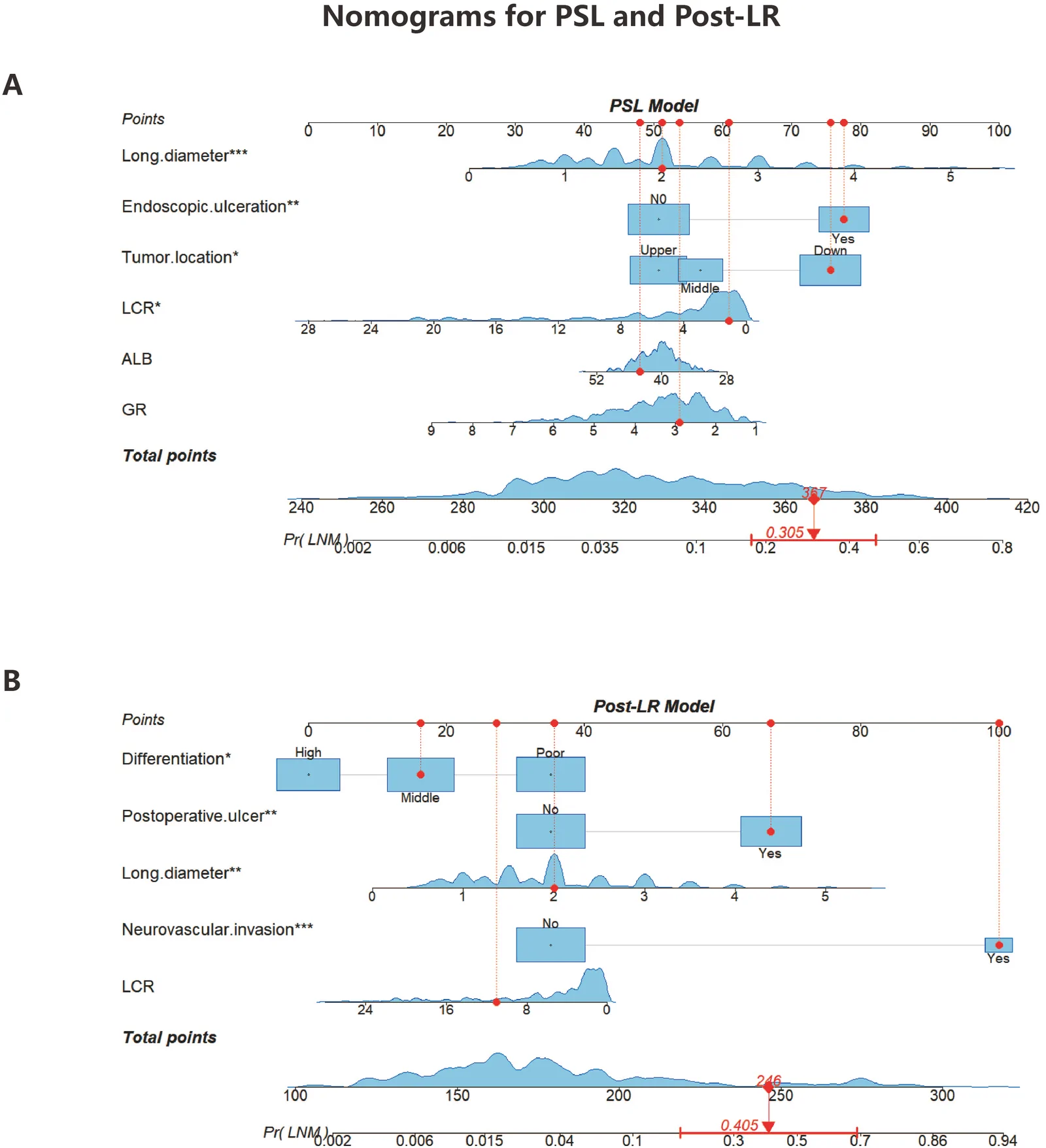
Nomograms for PSL and Post-LR. **(A)** PSL nomogram based on GR, ALB, continuous LCR, tumor location, endoscopic ulceration, and long diameter. **(B)** Post-LR nomogram based on continuous LCR, neurovascular invasion, long diameter, pathological ulceration, and differentiation. Full equations and coding are provided in **Supplementary Table S2**.

A nomogram was constructed from the final PSL coefficients to provide an individualized preoperative LNM probability (**Figure 5A**).

To use the nomogram, clinicians should first locate the specific value of each variable on its corresponding axis. For continuous variables (e.g., Long Diameter, GR, LCR, ALB), the exact numerical value is identified; for categorical variables (e.g., Endoscopic Ulceration), the specific category (e.g., Yes/No) is selected. A vertical line is then drawn upwards from each variable’s position to the top “Points” scale to obtain the individual score. These individual scores are summed to calculate the “Total Points”. Finally, a vertical line is projected downwards from the “Total Points” axis to the “Probability” axis to determine the predicted risk of LNM.

For illustration, training-cohort Subject 100 had GR 2.9 × 10^9^/L, ALB 44 g/L, LCR 1.11, a lower-third tumor, endoscopic ulceration, and a long diameter of 2 cm. The refitted PSL equation yielded an estimated LNM probability of 30.6%. The example is educational and does not define a treatment threshold.

A second nomogram was constructed for Post-LR (**Figure 5B**). Training-cohort Subject 20 had LCR 11, neurovascular invasion, a long diameter of 2 cm, pathological ulceration, and moderate differentiation. The refitted equation yielded an estimated LNM probability of 40.1%. This example illustrates calculation only; the model has not been validated for directing additional surgery after ESD.

### 7.6 Indirect predictor-set benchmark comparisons

**Figure 6:**
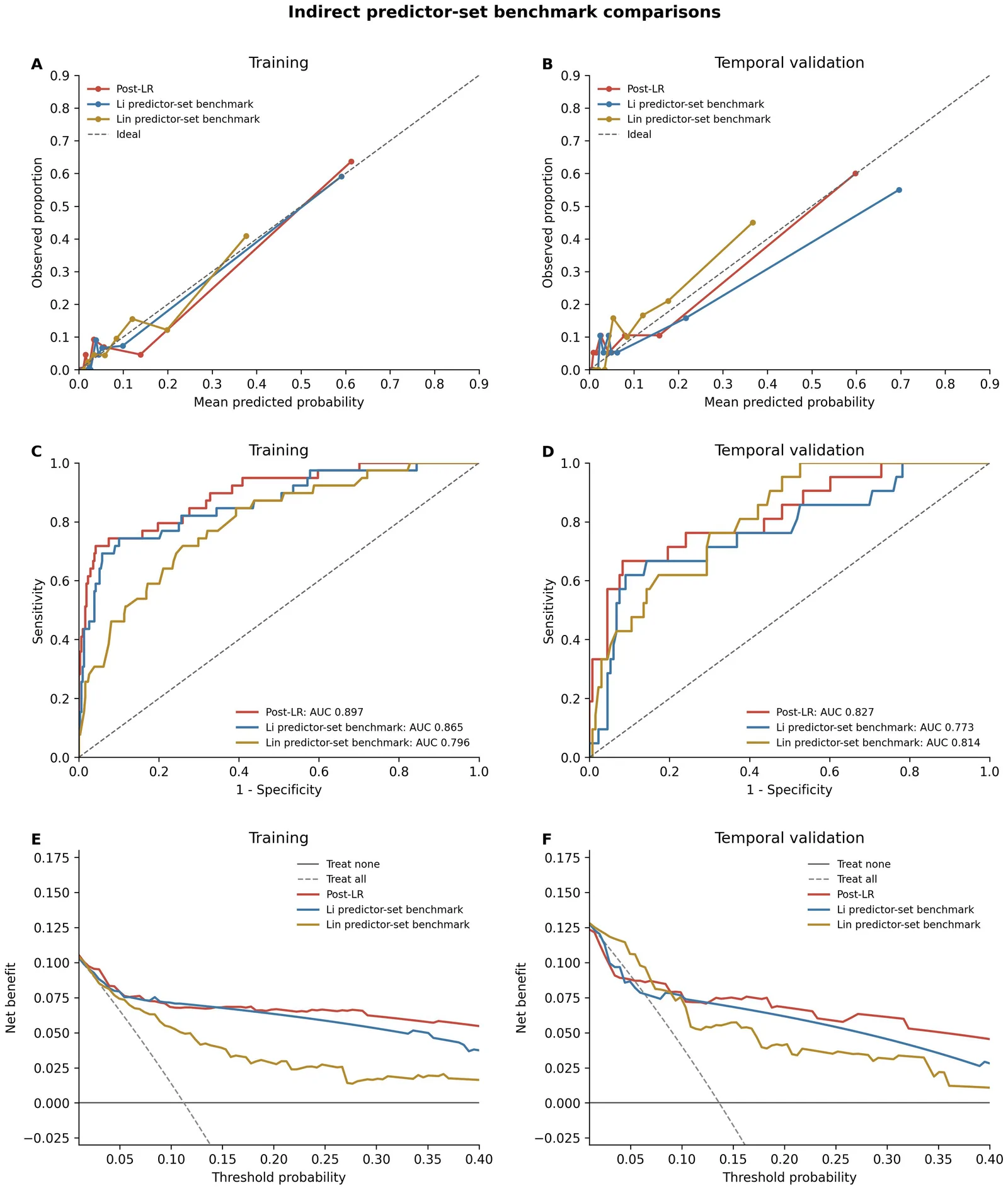
Indirect predictor-set benchmark comparisons. **(A, B)** Calibration plots in the training cohort **(A)** and temporal validation cohort **(B)**. **(C, D)** Receiver operating characteristic curves in the training cohort **(C)** and temporal validation cohort **(D)**. **(E, F)** Decision curve analyses in the training cohort **(E)** and temporal validation cohort **(F)**. Post-LR was compared with the predictor sets reported by Li et al. (19) and Lin et al. (20). Coefficients for all three specifications were estimated in the present training cohort and applied unchanged to temporal validation; thus, these comparisons do not constitute validation of the original published equations.

Post-LR was also compared with predictor sets reported by Li et al. (19) and Lin et al. (20). Because the original published equations and all required implementation details were not applied directly, the Li and Lin predictor sets were re-estimated in the present training cohort and then locked for temporal validation. These analyses are therefore indirect predictor-set benchmarks rather than external validations of the published models (**Figure 6**).

In the training cohort, Post-LR achieved an AUC of 0.897 (95% CI, 0.840–0.954), compared with 0.865 (95% CI, 0.796–0.933) for the Li predictor-set benchmark (P = 0.234) and 0.796 (95% CI, 0.722–0.870) for the Lin predictor-set benchmark (P = 0.005). In temporal validation, the AUC was 0.827 (95% CI, 0.723–0.932) for Post-LR, 0.773 (95% CI, 0.653–0.893) for the Li benchmark (P = 0.307), and 0.814 (95% CI, 0.727–0.900) for the Lin benchmark (P = 0.785).

Compared with the Lin benchmark, Post-LR improved NRI and IDI in the training cohort (NRI, 1.033; IDI, 0.275; both P < 0.001) and in temporal validation (NRI, 0.712; P = 0.003; IDI, 0.187; P = 0.002). Compared with the Li benchmark, improvements were observed in the training cohort (NRI, 0.582; P < 0.001; IDI, 0.074; P = 0.006) but not in temporal validation (NRI, 0.306; P = 0.204; IDI, 0.019; P = 0.555). These exploratory comparisons do not establish superiority over the original published models.

## 8 Exploratory analysis of an LCR cut-off

### 8.1 Training-derived LCR cut-off and temporal evaluation

**Figure 7:**
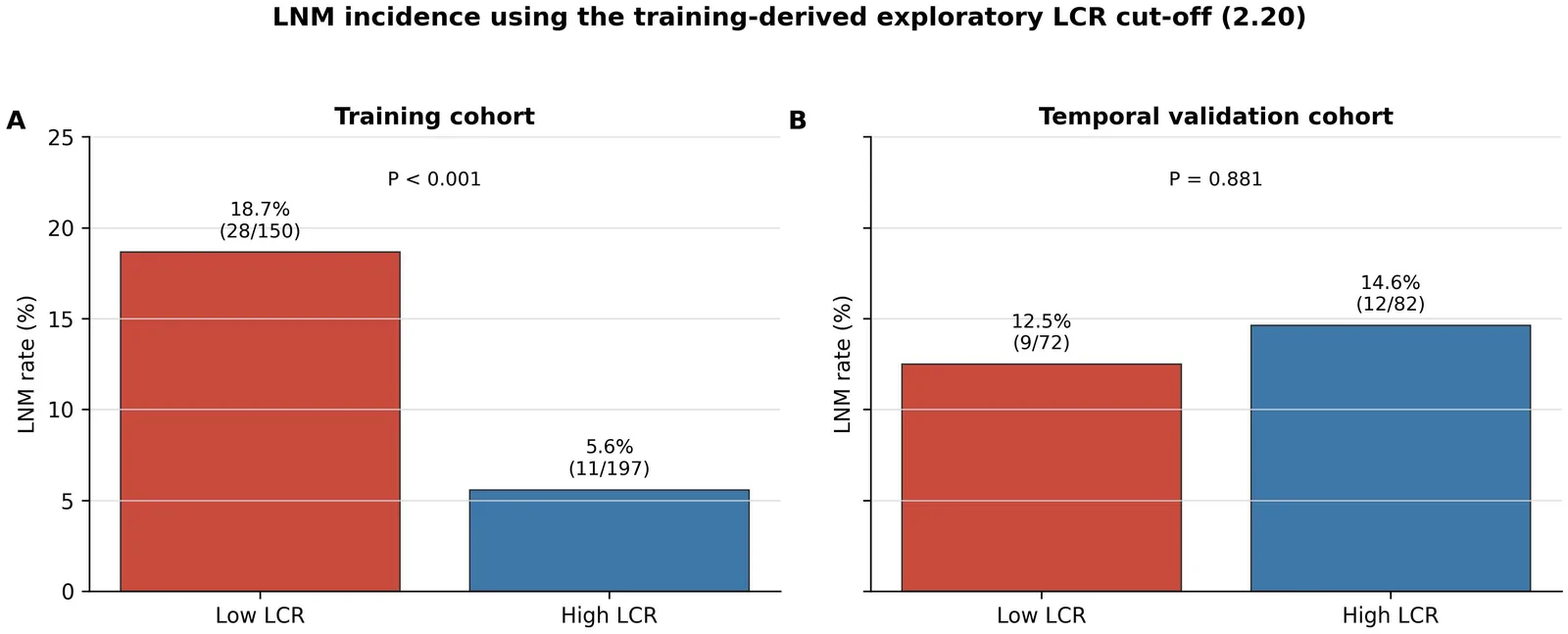
Exploratory LCR cut-off analysis. LNM incidence below and above the training-derived exploratory LCR cut-off of 2.20 in **(A)** the training cohort and **(B)** the temporal validation cohort. The cut-off separated risk only in training and should not be interpreted as a validated clinical threshold.

The minimum training-cohort chi-square P value occurred at the 44th percentile, corresponding to an LCR cut-off of 2.20. In the training cohort, LNM occurred in 28 of 150 patients (18.7%) with LCR < 2.20 and 11 of 197 (5.6%) with LCR ≥ 2.20 (P < 0.001). When the same cut-off was applied unchanged to the temporal validation cohort, LNM occurred in 9 of 72 patients (12.5%) and 12 of 82 patients (14.6%), respectively (P = 0.881). Thus, the training-derived cut-off did not transport to the later cohort (**Table 6**; **Figure 7**).

**Table 6:** LNM incidence using the training-derived exploratory LCR cut-off of 2.20.

Cohort	LCR group	Total	LNM positive	LNM rate	P
Training	Low (<2.20)	150	28	18.7%	<0.001
Training	High (≥2.20)	197	11	5.6%	
Temporal validation	Low (<2.20)	72	9	12.5%	0.881
Temporal validation	High (≥2.20)	82	12	14.6%	

The 2.20 cut-off was derived only in the training cohort and applied unchanged to temporal validation. P values are from chi-square tests. The cut-off scan was exploratory and was not adjusted for multiple testing.

### 8.2 Sensitivity analysis using categorized LCR

With the high-LCR group as the reference, low LCR was associated with LNM in the training cohort in univariable analysis (OR, 3.88; 95% CI, 1.86–8.08; P < 0.001) and after replacement of continuous LCR by the binary indicator in training-only sensitivity versions of PSL (OR, 5.52; 95% CI, 2.36–12.91; P < 0.001) and Post-LR (OR, 3.56; 95% CI, 1.39–9.11; P = 0.008). In temporal validation, the univariable association was absent (OR, 0.83; 95% CI, 0.33–2.11; P = 0.700). No validation-cohort model was refitted for this sensitivity analysis (**Table 7**).

**Table 7:** Sensitivity analysis of categorized LCR.

Cohort	Analysis	Comparison	OR (95% CI)	P
Training	Univariable	Low vs high LCR	3.88 (1.86–8.08)	<0.001
Training	PSL sensitivity refit	Low vs high LCR	5.52 (2.36–12.91)	<0.001
Training	Post-LR sensitivity refit	Low vs high LCR	3.56 (1.39–9.11)	0.008
Temporal validation	Univariable only	Low vs high LCR	0.83 (0.33–2.11)	0.700

Low LCR is compared with high LCR (reference). Adjusted models were refitted only in the training cohort after replacing continuous LCR with the binary indicator. No model was refitted in temporal validation.

These findings support retaining LCR as a continuous predictor in the primary models. The 2.20 cut-off should not be used as a clinical decision threshold without independent validation.

## 9 Model performance and clinical utility

Three locked models were compared: PSL, which used only preoperative variables; a pathology-only model comprising neurovascular invasion, differentiation, pathological ulceration, and pathological depth; and Post-LR, which combined preoperative and pathological variables. Full coefficients and coding are provided in **Supplementary Table S2**.

### 9.1 Discrimination

**Figure 8:**
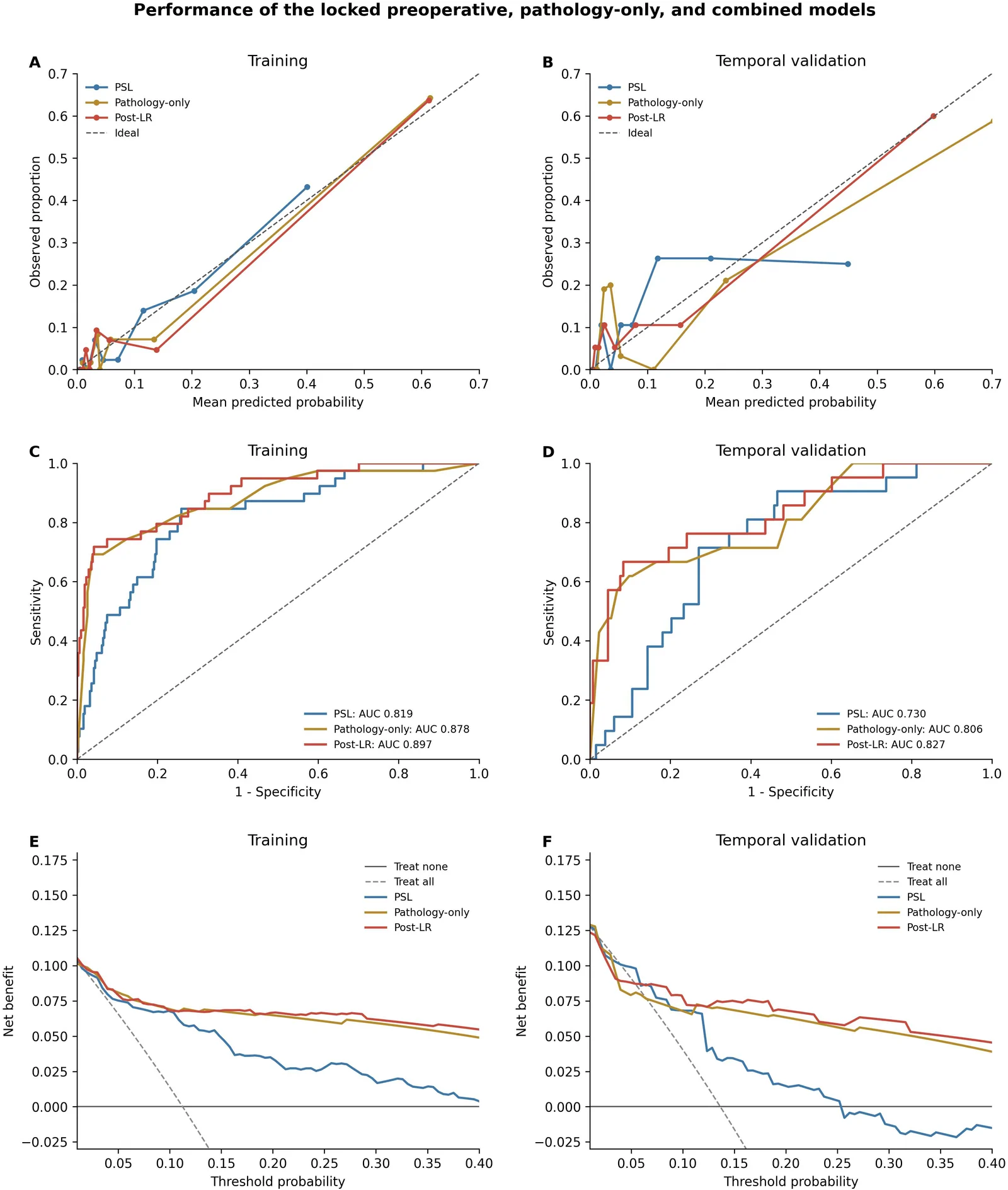
Performance of the locked final models. Calibration in **(A)** training and **(B)** temporal validation; ROC curves in **(C)** training and **(D)** temporal validation; and decision curves in **(E)** training and **(F)** temporal validation. PSL is the preoperative model, the pathology-only model uses only resection pathology variables, and Post-LR is the combined preoperative–pathological model.

Training-cohort AUCs were 0.819 (95% CI, 0.748–0.890) for PSL, 0.878 (95% CI, 0.811–0.944) for the pathology-only model, and 0.897 (95% CI, 0.840–0.954) for Post-LR. In temporal validation, the respective AUCs were 0.730 (95% CI, 0.627–0.834), 0.806 (95% CI, 0.698–0.915), and 0.827 (95% CI, 0.723–0.932) (**Table 8**; **Figures 8C, D**).

**Table 8:** Incremental predictive performance among PSL, the pathology-only model, and Post-LR.

Comparison	Cohort	AUC	AUC P	Continuous NRI (95% CI)	P	IDI (95% CI)	P
Pathology-only vs PSL	Training	0.878 vs 0.819	0.121	0.793 (0.492 to 1.070)	<0.001	0.218 (0.125 to 0.307)	<0.001
Post-LR vs PSL	Training	0.897 vs 0.819	0.011	1.116 (0.839 to 1.373)	<0.001	0.262 (0.173 to 0.343)	<0.001
Post-LR vs pathology-only	Training	0.897 vs 0.878	0.118	0.529 (0.196 to 0.844)	0.004	0.045 (0.008 to 0.082)	0.015
Pathology-only vs PSL	Temporal validation	0.806 vs 0.730	0.325	0.441 (0.010 to 0.887)	0.048	0.249 (0.106 to 0.394)	<0.001
Post-LR vs PSL	Temporal validation	0.827 vs 0.730	0.184	0.672 (0.231 to 1.093)	0.003	0.241 (0.118 to 0.364)	<0.001
Post-LR vs pathology-only	Temporal validation	0.827 vs 0.806	0.365	-0.010 (-0.441 to 0.446)	0.986	-0.008 (-0.052 to 0.034)	0.730

Positive NRI or IDI values favor the first model in each comparison. AUC P values are from DeLong’s test; NRI and IDI use 4,000 outcome-stratified bootstrap samples. PSL, preoperative model; Post-LR, combined preoperative–pathological model.

Relative to PSL, Post-LR improved training-cohort AUC (difference, 0.078; P = 0.011), NRI, and IDI. In temporal validation, NRI and IDI favored Post-LR, whereas the AUC difference was not significant (P = 0.184). The pathology-only model also improved NRI and IDI over PSL, although AUC differences were not significant in either cohort. Compared with the pathology-only model, Post-LR showed training-cohort improvements in NRI (0.529; P = 0.004) and IDI (0.045; P = 0.015), but its AUC difference was not significant. No incremental benefit of Post-LR over the pathology-only model was demonstrated in temporal validation (AUC P = 0.365; NRI P = 0.986; IDI P = 0.730).

### 9.2 Calibration

In the training cohort, all three refitted models had calibration intercepts near 0 and slopes near 1 by construction. In temporal validation, Post-LR had the calibration estimates closest to ideal (Brier score, 0.081; intercept, −0.079; slope, 0.783), whereas PSL showed greater overprediction and shrinkage (Brier score, 0.119; intercept, −0.625; slope, 0.544). The pathology-only model had a Brier score of 0.086, intercept of −0.329, and slope of 0.722. Hosmer–Lemeshow results were interpreted descriptively and did not override the calibration intercept, slope, and plots (**Table 9**; **Figures 8A, B**).

**Table 9:** Calibration performance of the locked final models.

Model	Cohort	Brier	Calibration intercept	Calibration slope	HL P
PSL	Training	0.082	-0.000	1.000	0.217
PSL	Temporal validation	0.119	-0.625	0.544	0.011
Pathology-only	Training	0.060	-0.000	1.000	0.393
Pathology-only	Temporal validation	0.086	-0.329	0.722	<0.001
Post-LR	Training	0.055	-0.000	1.000	0.335
Post-LR	Temporal validation	0.081	-0.079	0.783	0.085

Training values are apparent estimates from model refits. Temporal validation values were calculated without refitting or recalibration. Ideal calibration has intercept 0 and slope 1.

**Figure 9:**
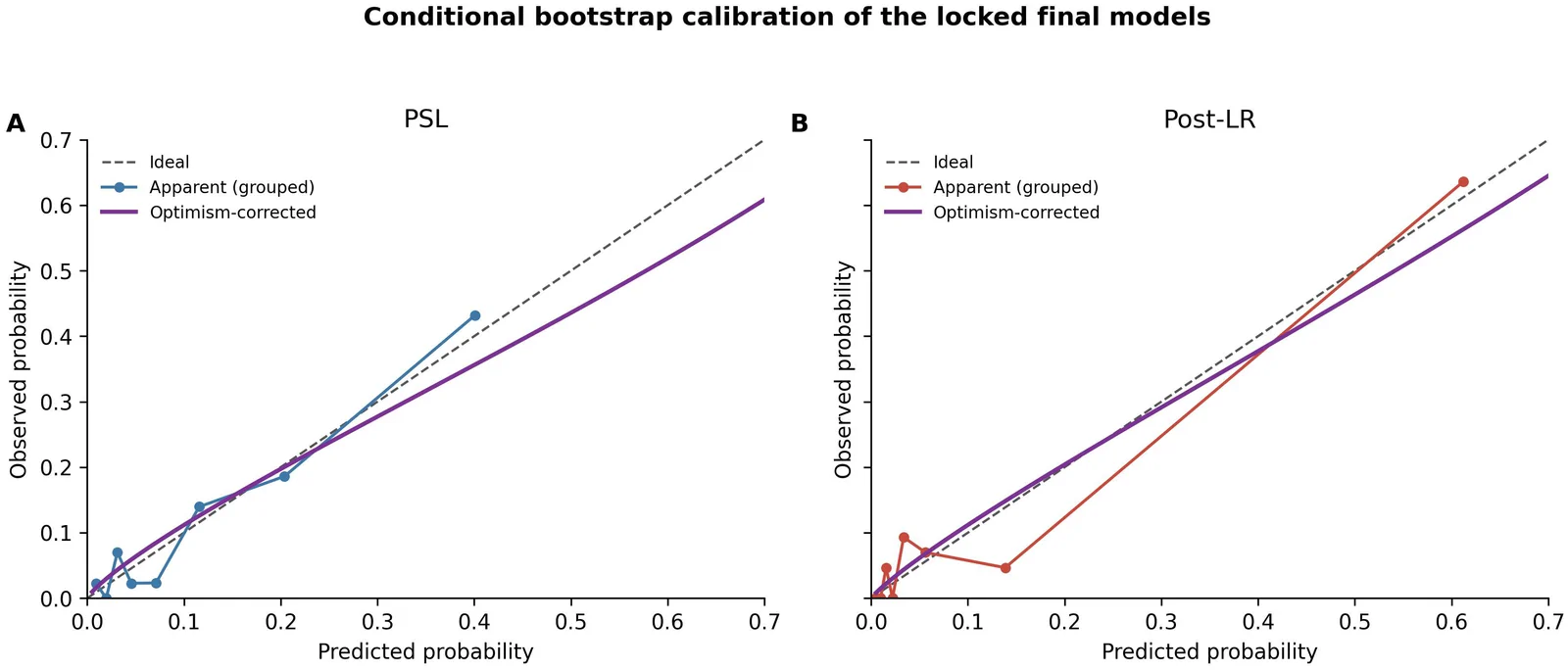
Conditional bootstrap calibration of PSL and Post-LR. Apparent grouped calibration and optimism-corrected logistic calibration for **(A)** PSL and **(B)** Post-LR in the training cohort. Predictor sets were fixed during 1,000 bootstrap repetitions; selection uncertainty is not represented.

Conditional bootstrap validation yielded an optimism-corrected C-index of 0.784 and Brier score of 0.089 for PSL, with a corrected calibration slope of 0.826. For Post-LR, the corresponding values were 0.874, 0.060, and 0.877. Because predictor selection was not repeated within each bootstrap sample, these estimates are conditional on the final specifications and may understate total model-development uncertainty (**Table 10**; **Figure 9**).

**Table 10:** Conditional bootstrap internal validation of PSL and Post-LR.

Metric	Apparent	Optimism/penalty	Optimism-corrected
PSL			
C-index	0.819	0.034	0.784
Brier score	0.082	0.007	0.089
Calibration slope	1.000	0.174	0.826
Calibration intercept	0.000	0.259	-0.259
Post-LR			
C-index	0.897	0.023	0.874
Brier score	0.055	0.005	0.060
Calibration slope	1.000	0.123	0.877
Calibration intercept	0.000	0.146	-0.146

One thousand bootstrap samples were used. The final predictor sets were held fixed; variable selection was not repeated. PSL used seven predictor parameters (39/7; EPV = 5.57), and Post-LR used six predictor parameters (39/6; EPV = 6.50). For Brier score, the reported optimism is the performance penalty added to the apparent value.

### 9.3 Clinical utility

Decision curves are shown in **Figures 8E, F**. Post-LR had the highest net benefit over much of the examined 0.01–0.40 threshold range, and the pathology-only model generally exceeded PSL in the temporal validation cohort. These curves should be interpreted as exploratory because the cohort was small, the models were derived from surgical patients, and no treatment-effect model was available.

Overall, pathological information added predictive information beyond the preoperative model. However, the temporal validation data did not demonstrate that adding preoperative variables to the pathology-only model further improved performance. The combined model should therefore be viewed as a candidate post-resection risk tool rather than a validated replacement for established curability criteria.

## 10 Discussion

Accurate LNM assessment remains central to choosing between endoscopic and surgical treatment in EGC (21, 22). PSL and Post-LR address different time points, but neither should replace guideline-based assessment. A valid head-to-head comparison with standard ESD or eCura criteria could not be reconstructed from this dataset. In particular, the database did not contain the complete pre-ESD biopsy histology/differentiation, clinical depth assessment (including endoscopic ultrasonography where applicable), en bloc resection status, horizontal and vertical margin status, or separately recorded lymphatic and venous invasion required to reproduce those standards. The preoperative model also does not include differentiation or clinical depth. Consequently, a direct accuracy comparison would use a non-equivalent reference and was not performed.

PSL uses routine preoperative laboratory and endoscopic variables, whereas Post-LR adds neurovascular invasion, pathological ulceration, and differentiation. The latter information becomes available only after resection. The model comparison therefore evaluates incremental prediction within this surgical dataset, not whether the nomograms outperform ESD guidelines. Likewise, the Li and Lin analyses are predictor-set benchmarks with locally re-estimated coefficients and should be interpreted as indirect comparisons.

### 10.1 Discussion of predictive factors

Systemic inflammation may contribute to tumor progression, and lower LCR has been associated with adverse outcomes in gastric cancer (23–25). In the present study, continuous LCR was retained in both final models and was associated with lower odds of LNM in PSL. The exploratory cut-off of 2.20 separated training-cohort risk but failed in temporal validation, and categorical LCR was not associated with LNM in group 2. This instability, together with the data-driven threshold scan, argues against presenting 2.20 as a clinically established cut-off. GR and ALB were retained in PSL as part of the training-derived multivariable specification, but neither coefficient was individually significant; their contributions should therefore be interpreted at the model level rather than as confirmed independent risk factors (26, 27).

Similarly, lesion size emerged as a robust predictor in both models. A larger tumor diameter increases the opportunity for lymphatic involvement, consistent with prior EGC studies identifying size as an important predictor of LNM (28–34). Tumor location was also retained in the preoperative model. Middle- and lower-third cancers may differ in LNM risk (35), and anatomical location has been reported as an independent predictor (14, 36), plausibly reflecting differences in lymphatic drainage (37, 38). These observations support including size and location in preoperative risk estimation, while the present retrospective analysis does not establish causality.

Endoscopic and pathological ulceration represent related but non-identical observations. Endoscopic assessment can be subjective and may disagree with histology, whereas pathological ulceration is defined from the resected specimen (39, 40). In this study, endoscopic ulceration was eligible for the preoperative model and pathological ulceration for the combined and pathology-only models. Their inclusion is consistent with the role of ulceration in established endoscopic curability frameworks, but it does not by itself establish a causal mechanism (5, 39–43).

Neurovascular invasion and differentiation were retained in Post-LR. Neurovascular invasion had the largest adjusted association with LNM, consistent with evidence that lymphovascular or perineural invasion is closely related to nodal spread in gastric cancer (44–49). Relative to poor differentiation, well differentiation was associated with lower odds in the combined model, whereas the moderate category was not statistically significant. These estimates are imprecise because only 39 LNM events were available for model development (42, 45, 50).

The final combined model therefore captures complementary systemic, endoscopic, and pathological information. Nevertheless, the failure to demonstrate incremental value over the pathology-only model in temporal validation suggests that the pathological variables carry much of the predictive signal. Pathological ulceration should be interpreted as a standardized histological feature relevant to endoscopic curability assessment, not as proof of an unmeasured biological pathway (39, 40, 43, 51).

### 10.2 Clinical application and accessibility

Nomograms were produced to illustrate the two equations, and worked examples are provided in Section 7.5. The web calculators reported by the authors are https://nomogram-egc.shinyapps.io/Preoperative-Nomogram-for-EGC and https://nomogram-egc.shinyapps.io/Postoperative-Nomogram-for-EGC. Their functionality and agreement with the coefficients in **Supplementary Table 2** should be verified immediately before submission. Post-LR is intended only for exploratory risk re-evaluation after ESD; it should not be used to recommend additional gastrectomy without guideline assessment and multidisciplinary review.

### 10.3 Model performance and incremental value

The comparison among PSL, the pathology-only model, and Post-LR demonstrates that pathological data add information beyond a preoperative model. It does not establish that a combined model is necessary in every setting. In particular, Post-LR did not outperform the pathology-only model in temporal validation. Future work should test whether the preoperative components add clinically meaningful value after ESD pathology is known, using prespecified models and independent cohorts.

### 10.4 Limitations and future directions

This study has several limitations. First, all patients underwent radical gastrectomy with lymphadenectomy rather than ESD alone; therefore, the intended post-ESD use of Post-LR is indirect and subject to spectrum and treatment-selection bias. Second, the database lacked several variables required to reconstruct standard ESD/eCura criteria, including complete pre-ESD biopsy differentiation, clinical invasion depth, en bloc resection, horizontal and vertical margins, and separately documented lymphatic and venous invasion. A valid head-to-head guideline comparison was therefore not possible. Third, the study was retrospective and single-center. The time split provides temporal internal validation but not geographic or institutional external validation. Fourth, only 39 LNM events were available in training and 21 in validation. PSL used seven predictor parameters (EPV = 39/7 = 5.57) and Post-LR used six (EPV = 39/6 = 6.50), increasing the risk of unstable coefficients and optimistic performance. The fixed-predictor bootstrap did not repeat the entire selection process and may underestimate model-development uncertainty. Fifth, the LCR cut-off was selected after scanning multiple training thresholds, was not adjusted for multiplicity, and failed temporal validation. Finally, comparisons with published models used locally re-estimated predictor sets rather than the original equations. Prospective multicenter validation in a real-world ESD cohort, with standardized collection of guideline variables and prespecified analysis, is required.

## 11 Conclusion

We developed a preoperative model and a combined preoperative–pathological model for LNM in EGC. Both showed useful discrimination, but temporal validation revealed calibration limitations, and the combined model did not demonstrate incremental value over a pathology-only model in the later cohort. The exploratory LCR cut-off did not validate. These nomograms should therefore be regarded as adjunctive research tools rather than stand-alone treatment rules. Independent prospective validation in ESD populations is necessary before clinical implementation.

## Data Availability

The raw data supporting the conclusions of this article will be made available by the authors, without undue reservation.

## Supplementary material

Supplementary Table 1 Stratified LNM incidence for key risk factors. P values compare categories within each cohort using Pearson chi-square tests (Yates correction for 2 × 2 tables). The LCR cut-off was derived in training only. LNM, lymph node metastasis.

Supplementary Table 2 Full model coefficients and coding. Predicted probability = 1/[1 + exp(−linear predictor)]. Continuous variables use their original units. Binary variables are coded 1 = present and 0 = absent. Tumor location uses the upper third as reference; differentiation uses poor differentiation as reference. PSL contains seven predictor parameters because tumor location contributes two indicators. Post-LR contains six predictor parameters because differentiation contributes two indicators.
