# Supplementary material for: Development and temporal validation of preoperative and combined preoperative–pathological models for lymph node metastasis in early gastric cancer
Source: Front Oncol. 2026 Sep 11;16:1871868. doi: 10.3389/fonc.2026.1871868 (PMC13612299; doi:10.3389/fonc.2026.1871868)
Supplement: Supplementary file 1 [file Table1.pdf]

Supplementary Table S1. Stratified LNM incidence for key risk factors

Risk factor	Category	Training: LNM/total (rate)	P	Temporal validation: LNM/total (rate)	P
Tumor location	Upper third	6/123 (4.9%)	0.003	3/43 (7.0%)	0.035
	Middle third	7/79 (8.9%)		2/34 (5.9%)	
	Lower third	26/145 (17.9%)		16/77 (20.8%)	
Endoscopic ulceration	No	12/206 (5.8%)	<0.001	10/96 (10.4%)	0.209
	Yes	27/141 (19.1%)		11/58 (19.0%)	
Neurovascular invasion	No	12/300 (4.0%)	<0.001	8/127 (6.3%)	<0.001
	Yes	27/47 (57.4%)		13/27 (48.1%)	
Long diameter	≤2 cm	15/236 (6.4%)	<0.001	11/101 (10.9%)	0.261
	>2 cm	24/111 (21.6%)		10/53 (18.9%)	
Tumor depth	T1a	8/147 (5.4%)	0.006	2/66 (3.0%)	0.002
	T1b	31/200 (15.5%)		19/88 (21.6%)	
Pathological ulceration	No	10/196 (5.1%)	<0.001	9/82 (11.0%)	0.429
	Yes	29/151 (19.2%)		12/72 (16.7%)	
Differentiation	Poor	26/123 (21.1%)	<0.001	16/50 (32.0%)	<0.001
	Moderate	10/117 (8.5%)		5/54 (9.3%)	
	Well	3/107 (2.8%)		0/50 (0.0%)	
LCR group	Low (<2.20)	28/150 (18.7%)	<0.001	9/72 (12.5%)	0.881
	High (≥2.20)	11/197 (5.6%)		12/82 (14.6%)	

Note: P values compare categories within each cohort using Pearson chi-square tests (Yates correction for 2 × 2 tables). The LCR cut-off was derived in training only. LNM, lymph node metastasis.
