# Supplementary material for: Development and temporal validation of preoperative and combined preoperative–pathological models for lymph node metastasis in early gastric cancer
Source: Front Oncol. 2026 Sep 11;16:1871868. doi: 10.3389/fonc.2026.1871868 (PMC13612299; doi:10.3389/fonc.2026.1871868)
Supplement: Supplementary file 2 [file Table2.pdf]

Supplementary Table S2. Full model coefficients and coding

Model	Term	β	OR	95% CI	P
PSL	Intercept	-1.99832	0.136	0.002–11.164	0.375
PSL	GR	-0.27793	0.757	0.536–1.071	0.116
PSL	ALB	-0.03757	0.963	0.868–1.068	0.477
PSL	LCR	-0.10768	0.898	0.821–0.982	0.018
PSL	Tumor location: Middle third vs Upper third	0.28293	1.327	0.397–4.439	0.646
PSL	Tumor location: Lower third vs Upper third	1.17475	3.237	1.196–8.765	0.021
PSL	Endoscopic ulceration	1.26702	3.550	1.604–7.856	0.002
PSL	Long diameter	0.65783	1.931	1.349–2.762	<0.001
Pathology-only	Intercept	-3.41191	0.033	0.011–0.097	<0.001
Pathology-only	Neurovascular invasion	3.04318	20.972	8.385–52.450	<0.001
Pathology-only	Differentiation: Moderate vs Poor	-0.79821	0.450	0.171–1.186	0.106
Pathology-only	Differentiation: Well vs Poor	-1.27717	0.279	0.072–1.083	0.065
Pathology-only	Pathological ulceration	1.32896	3.777	1.524–9.364	0.004
Pathology-only	Tumor depth	0.17083	1.186	0.441–3.191	0.735
Post-LR	Intercept	-4.18930	0.015	0.003–0.066	<0.001
Post-LR	LCR	-0.06492	0.937	0.857–1.025	0.157
Post-LR	Neurovascular invasion	2.80727	16.565	6.777–40.490	<0.001
Post-LR	Long diameter	0.56740	1.764	1.150–2.706	0.009
Post-LR	Pathological ulceration	1.38261	3.985	1.543–10.292	0.004
Post-LR	Differentiation: Moderate vs Poor	-0.82090	0.440	0.164–1.181	0.103
Post-LR	Differentiation: Well vs Poor	-1.52452	0.218	0.051–0.927	0.039

Note: Predicted probability = 1/[1 + exp(−linear predictor)]. Continuous variables use their original units. Binary variables are coded 1 = present and 0 = absent. Tumor location uses the upper third as reference; differentiation uses poor differentiation as reference. PSL contains seven predictor parameters because tumor location contributes two indicators. Post-LR contains six predictor parameters because differentiation contributes two indicators.
